# Differences in Cause-Specific Mortality Between Frail Men and Women in the United States

**DOI:** 10.1093/geroni/igaa057.868

**Published:** 2020-12-16

**Authors:** Matthew Lohman, Amanda Sonnega, Amanda Leggett, Nicholas Resciniti

**Affiliations:** 1 University of South Carolina, Columbia, South Carolina, United States; 2 University of Michigan, Ann Arbor, Michigan, United States

## Abstract

While frailty is associated with risk of numerous adverse health outcomes including mortality, little is known about the most common specific causes of death among frail older adults or how these causes might differ by gender. This information may be important to understanding the frailty syndrome and to informing screening and treatment. We used linked data from the Health and Retirement Study (2004 – 2012) and the National Death Index (NDI). We analyzed data from HRS participants age 65 and older who completed a general health interview and physiological measures (n=10,490). Frailty was operationalized using the phenotype criteria – low weight, low energy expenditure, exhaustion, slow gait, and weakness. Causes of death were determined using International Classification of Diseases (v10) codes from death certificates. We used Cox proportional hazards to compare incidence of cause-specific mortality by frailty status and gender. The attributable risk of mortality due to frailty in the sample was 16.6% among women and 17.3% among men. Overall, frail older adults had greater risk of death from heart disease (hazard ratio (HR): 2.97; 95% CI: 2.18, 4.04), cancer (HR: 2.81; 95% CI: 2.01, 3.93), and dementia 2.86 (95% CI: 1.46, 5.58) but not cerebrovascular disease or accidents. Frail women were more approximately 29% more likely to die from heart disease than frail men. Findings suggest that frailty is a significant risk factor for mortality from several different causes, especially among women. Findings may help inform screening and treatment decisions for older adults at risk for frailty.

